# Characterization of an Antihypertensive Angiotensin I-Converting Enzyme Inhibitory Peptide from the Edible Mushroom *Hypsizygus marmoreus*

**DOI:** 10.1155/2013/283964

**Published:** 2013-11-27

**Authors:** Min-Gu Kang, Young-Hun Kim, Zanabaatar Bolormaa, Min-Kyung Kim, Geon-Sik Seo, Jong-Soo Lee

**Affiliations:** ^1^Department of Biomedicinal Science and Biotechnology, PaiChai University, Daejeon 302-735, Republic of Korea; ^2^Korea National College of Agricultural and Fishery, Hwasung, Kyonggi-do 445-893, Republic of Korea

## Abstract

Hypertension is one of the very serious diseases and, recently, hypertensive patient longevity has been increased significantly. Therefore, the development of new antihypertensive drugs or bioactive compounds is very important to remedy or prevent hypertension. The antihypertensive angiotensin I-converting enzyme (ACE) inhibitor in water extracts from the brown-cultivar-fruiting-body of *Hypsizygus marmoreus* was purified with ultrafiltration, C_18_ solid phase extraction chromatography and reverse-phase HPLC, and the purified ACE inhibitor with inhibitory activity of IC_50_ value of 0.19 mg/mL was obtained. The purified ACE inhibitor was found to be a new oligopeptide with the sequence LSMGSASLSP. Its molecular weight was estimated to be 567.3 Da and the water extracts containing ACE inhibitor from *Hypsizygus marmoreus* showed a clear antihypertensive action a spontaneously hypertensive rat.

## 1. Introduction

*Hypsizygus marmoreus* (family Tricholomataceae) is an edible fungus (Basidiomycetes) with a delicious taste and unique texture. It is found in Korea, Japan, China, North Europe, and East Asia. It generally grows well in the stumps of beech, maple, and blighted trees. Recent studies have demonstrated that this species provides antitumor and antioxidant effects. Its antitumor polysaccharide, *β*-(1-3)-D-glucan has an anticancer activity [1]. Mori et al. [2] reported that a dietary supplement containing *H. marmoreus* powder lowered total serum cholesterol and had a strong antiatherosclerotic effect. There was also an antioxidant effect [3, 4], and *β*-(1-3)-D-glucan isolated from *H. marmoreus* showed very high antitumor activity [5].

Many antihypertensive, angiotensin I-converting enzyme (dipeptidyl carboxy peptidase I, kinase II, E.C 3.4.15.1, ACE) inhibitors have been identified in various microorganisms including *Saccharomyces cerevisiae* [6], *Grifola frondosa* [7], *Ganoderma lucidum* [8], *Tricholoma giganteum* [9], *Pholiota adiposa* [10], and *Pleurotus cornucopiae* [11], ACE inhibitors have also been isolated from food and the enzymatic digestives of food proteins including, gelatin, casein, fish, fig tree latex, a-zein [12], sake and its byproducts [13], Korean traditional rice wines and liquors [14], and cereals and legumes [15]. Although many natural and synthetic ACE inhibitors (e.g., captopril, enalapril, and lisinopril), are effective as antihypertensive drugs, they also have some disadvantages, such as easy digestion by protease in the body, and side effects, such as coughing, allergies, taste disturbances, and skin rashes [6]. Therefore, the development of new ACE inhibitors that have strong antihypertensive activity, and resistance to digestion by various proteases; without side effects, is necessary. In a previous paper [16], we reported on the production of *Hypsizygus marmoreus*. In this study, an ACE inhibitor from the brown-cultivar-fruiting-body of *H. marmoreus* was purified and characterized.

## 2. Methods

### 2.1. Preparation of *Hypsizygus marmoreus* Extracts

Dried fruiting bodies (50 g) of *H. marmoreus* (brown cultivar) containing antihypertensive ACE inhibitor were pulverized, added to 1.5 L water, and shaken at 50°C for 12 h. The mixtures was centrifuged at 5000 **×**g for 30 min and filtered with a Whatman No. 41 filter paper and 0.45 *μ*m pore size filter (Nalgene, USA). The supernatant was lyophilized and used as a water extract.

### 2.2. Assay of ACE Inhibitory Activity

The ACE inhibitory activity was assayed by the modified method of Cushman and Cheung [17]. A mixture containing 100 mM sodium borate buffer (pH 8.3), 300 mM NaCl, 150 *μ*L (3 units) of ACE from rabbit lungs, and 50 *μ*L of sample solution was preincubated for 10 min at 37°C. The reaction was initiated by adding 50 *μ*L of Hip-His-Leu at a final concentration of 5 mM. It was terminated after 30 min of incubation by the addition of 250 *μ*L of 1.0 M HCl. The liberated hippuric acid was extracted with 1 mL of ethyl acetate, and 0.8 mL of the extract was evaporated using a Speed Vac Concentrator (EYELA Co., Japan). The residue was then dissolved in 1 mL of sodium borate buffer. Absorbance at 228 nm was measured to estimate the ACE inhibitory activity. The inhibition activity was calculated using

(1) $$\begin{array}{c} \text{inhibition}\;\text{activity}\;\left(\%\right)=\left(1-\frac{A-B}{C-D}\right)\times 100, \end{array}$$

where *A* is the absorbance of the solution containing ACE, substrate and sample, *B* is the absorbance of the solution containing ACE and sample without the substrate, *C* is the absorbance of the solution containing ACE and substrate without the sample, and *D* is the absorbance of the solution containing only substrate.

The concentration of the ACE inhibitor required to inhibit 50% of the ACE activity under the above assay condition was defined as IC_50_.

### 2.3. Purification of ACE Inhibitor

The water-extract solution was subjected to ultrafiltrate in with 50,000 and 5,000 M.W. cutoff filters (Labscale TFF System, Millipore Co., USA), and the ACE inhibitory activities of the filtrates and solutions of the filter-cake were determined. The active fraction was treated with three kinds of proteases (pepsin, trypsin, and pancreatin). The active fraction was lyophilized and applied to a C_18_ solid-phase extraction (Sep-Pak C_18_ Cartridges, Waters Co., Milford, MA, USA), equilibrated with 5% acetonitrile. A gradient was carried out in water from 5%, 25%, 50% and 100%. The active fraction was lyophilized and it was applied to a strong cation exchange (SCX), solid-phase, extraction process (Hypersep SCX, Thermo Scientific Co., MA, USA), equilibrated with 10 mM ammonium formate, and eluted with ammonium formate (10, 30, 50, 100, and 200 mM). The active fraction obtained was then applied to reverse-phase, high-performance, liquid chromatography (RP-HPLC) (Vydac 218TP54, C_18_ column, 5 *μ*m, 4.6 × 250 mm, Discovery Science Co., USA), equilibrated with 5% acetonitrile. A linear gradient (from 5 to 25% water) was carried out with 0.1% trifluoroacetic acid (TFA). The active fractions were collected and lyophilized immediately.

### 2.4. Antihypertensive Action of the Purified Ace Inhibitor

Spontaneously hypertensive male rats (SHR, SHR/NCrljOri) of body weight 190–220 g and nine weeks old were purchased from Samtaco Bio-Korea Co. (Korea, Osan City). SHRs were housed individually in steel cages in a room at 24°C with a 12 h light-dark cycle, and fed a standard diet. Tap water was freely available. Water extract of the *H. marmoreus* fruiting body was dissolved in saline at a dose of 800 mg/kg body weight and injected orally in SHRs. The systolic blood pressure of the animals was measured before and after 0, 2, 4, 6, and 8 h of administration by the rat tail-cuff method using a specially devised Blood Pressure Monitoring System (CODA Monitor, Kent Scientific Co., Torrington, CT, USA).

Each experimental group consisted of five SHRs. Negative and positive control groups were also used. The positive control group was administered the commercial antihypertensive drug Captopril (ACE inhibitor), at a dose of 100 mg/kg, whereas saline was administered to the negative control group. Prior to treatment of the SHRs, blood was measured. While the ACE inhibitor was being administered, the blood pressure of members of each group was measured three times during every test.

## 3. Results

### 3.1. Purification of the ACE Inhibitor

After the water extracts of *H. marmoreus *(brown cultivar) were ultrafiltered with a 5000 M.W. cutoff filter, the ACE inhibitory activity of two filtrates were then determined. The ACE inhibitory activity of 5000 M.W. over filtrates was 7.1 mg/mL and 5000 M.W. below filtrates showed 6.4 mg/mL of IC_50_ ACE inhibitory activity. When the active 5000 M.W. below filtrates was treated with some proteases, pepsin treatment resulted in higher ACE inhibitory activity (IC_50_; 4.3 mg/mL) than the other trypsin treatment (IC_50_; 8.7 mg/mL) and pancreatin treatment (IC_50_; 20.0 mg/mL); data is not shown. The effect of pepsin treatment on ACE inhibitor in this study was similar to that of ACE inhibitor from *Saccharomyces cerevisiae *[6].

**Figure 1:**
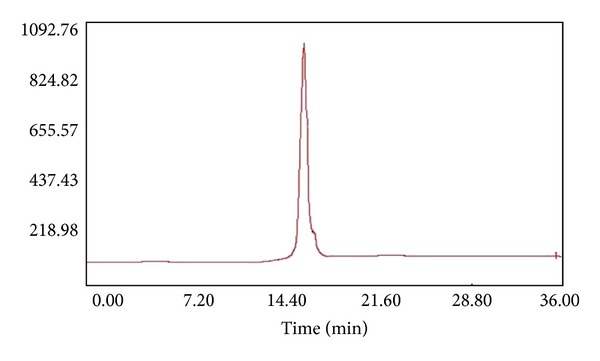
RP-HPLC chromatogram on l Vydac protein/peptide reverse-phase 218TP column of active fraction F1-2-3 (50 mM). Separation was performed with a linear gradient of acetonitrile containing 0.1% TFA from 5% to 25% at a flow rate of 1 mL/min.

After C_18_ solid-phase extraction from 5% to 100% using acetonitrile, the fraction F1-2 from the extraction with 25% acetonitrile showed high IC_50_: 0.57 mg/mL of ACE inhibitory activity. After SCX solid-phase extraction of the active fraction by 10–200 mM of ammonium formate, the active fraction (F1-2-1) from an extraction of 50 mM ammonium formate, 0.36 mg/mL of IC_50_ was obtained (data not shown). Subsequently, RP-HPLCs were performed on the active fraction the using a l-Vydac-protein/peptide reverse-phase 218TP column and a purified ACE inhibitor showing ACE inhibitory activity (IC_50_) of 0.34 mg/mL was obtained (Figure 1).

The inhibitory activity of the purified ACE inhibitor was lower than those of the mushrooms, *Tricholoma giganteum *(IC_50_ 0.04 mg/mL) [9],* Pholiota adiposa* ASI 24012 (0.044 mg/mL) [10], yeast: *Saccharomyces cerevisiae* (0.07 mg/mL) and captopril, an antihypertensive drug which was chemically synthesized (IC_50_: 0.004 mg/mL) [9].

### 3.2. Analysis of Amino Acid Sequence and Molecular Weight

**Figure 2:**
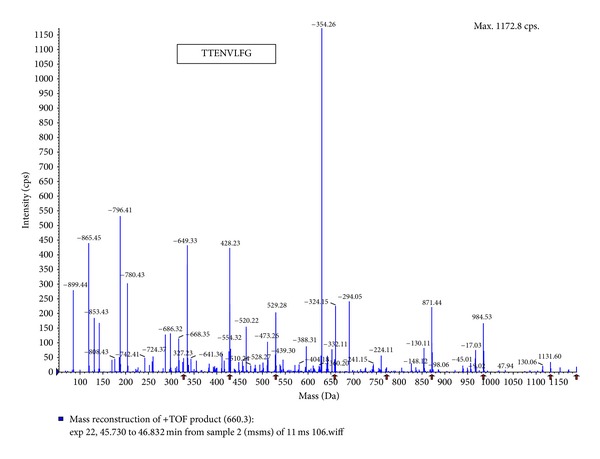
Amino acid sequence of the purified oligopeptide (P-1) from *H. marmoreus* by LC-MS/MS. MS/MS experiments were performed on a LCQ-Deca ESI ion trap mass spectrometer (Thermo Finnigan Co., USA). For protein identification, the MS/MS spectra were searched using SEQUEST (ver 3.3) software. (P-1, TTENVLFG).

**Figure 3:**
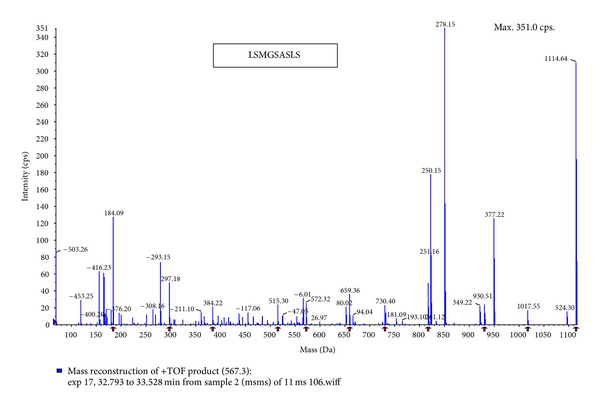
Amino acid sequence of the purified oligopeptide (P-2) from *H. marmoreus* by LC-MS/MS. MS/MS experiments were performed on a LCQ-Deca ESI ion trap mass spectrometer (Thermo Finnigan Co., USA). For protein identification, the MS/MS spectra were searched using SEQUEST (ver 3.3) software. (P-2, LSMGSASLSP).

**Figure 4:**
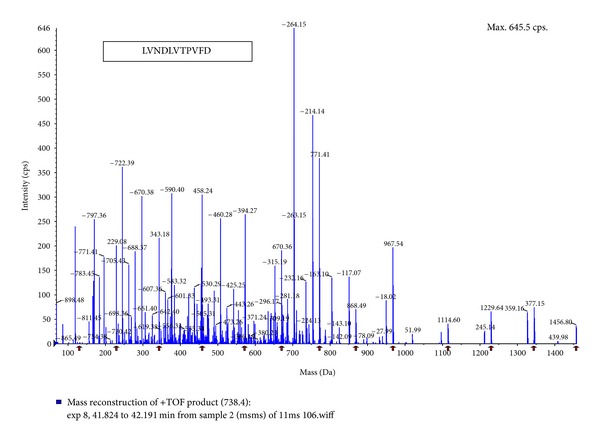
Amino acid sequence of the purified oligopeptide (P-3) from *H. marmoreus* by LC-MS/MS. MS/MS experiments were performed on a LCQ-Deca ESI ion trap mass spectrometer (Thermo Finnigan Co., USA). For protein identification, the MS/MS spectra were searched using SEQUEST (ver 3.3) software. (P-3, LVNDLVTPVFDNL).

The purified ACE inhibitor was analyzed by LC-MS/MS and three kinds of oligopeptide (i.e., TTENVLFG (P-1), LSMGSASLSP (P-2) and LVNDLVTPVFDNL (P-3)) were obtained (Figures 2, 3, and 4). After chemically synthesizing these three oligopeptides, their ACE inhibitory activities were determined. Chemically synthesized oligopeptides had inhibitory activity (IC_50_) of 3.03 mg/mL (P-1), 0.19 mg/mL (P-2) and 4.00 mg/mL (P-3), respectively. Thus, we successfully identified the P-2 oligopeptide as the purified ACE inhibitor.

Meanwhile, the molecular weight of the purified ACE inhibitor P-2 was estimated to be 567.3 Da without any homology. Its molecular weight was also similar to, or smaller than, those of the other ACE inhibitors from mushrooms *Pholiota adiposa* (414.0 Da) [10], *Pleurotus cornucopiae* (1622.85 Da, 2037.26 Da) [11], and *Tricholoma giganteum* (301.10 Da) [9]. See Table 1.

### 3.3. Determination of ACE Inhibition Pattern

**Figure 5:**
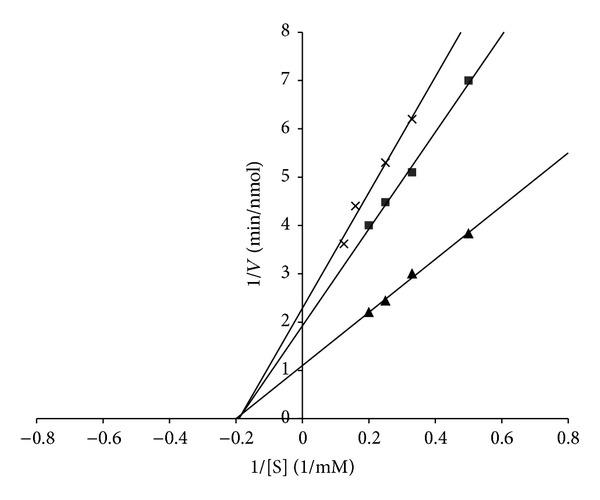
Lineweaver-Burk plot of ACE activity in the presence of the purified ACE inhibitor (P-2). (▲; Control, ■; 0.1 mg of inhibitor, ×; 0.3 mg of inhibitor).

The inhibitory pattern of the purified ACE inhibitor P-2 was investigated using a Lineweaver-Burk plot (Figure 5). It was found that the purified ACE inhibitor had a noncompetitive inhibitory pattern, unlike those of *Grifola frondosa *[7] and *Tricholoma giganteum* [9], which showed patterns of competitive inhibition.

### 3.4. Antihypertensive Action of the Purified Ace Inhibitor

The antihypertensive action of water-extracts from *H. marmoreus* was investigated using spontaneously hypertensive rats (SHRs).

**Figure 6:**
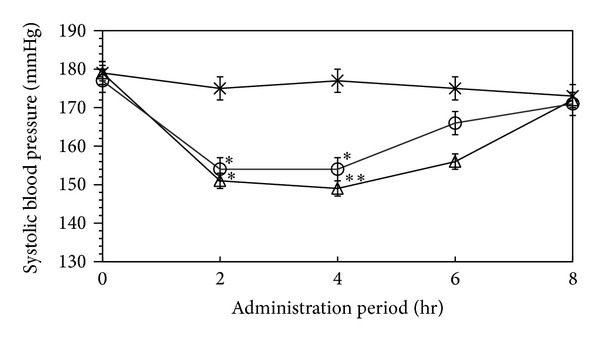
Changes in systolic blood pressure (SBP) of spontaneous hypertensive rat by administering water extract of *Hypsizygus marmoreus*. Single oral administration was performed with a dosage of 800 mg/Kg body weight, and SBP was measured after 0, 2, 4, and 6 h administration. Different from control at *P < 0.05, **P < 0.01. ×, Saline solution; △, Commercial captopril; ○, Water extract of *Hypsizygus marmoreus *containing antihypertensive angiotensin I-converting enzyme inhibitor.

As shown in Figure 6, the average blood pressure of the SHRs in the test group was approximately 180 mmHg just before the administration of the water extracts containing ACE inhibitor. Two hours after administration to the rats of the water extract (dosage 800 mg/kg), their blood pressure decreased to 154 mmHg, and after 4 h, the average blood pressure increased to 166 mmHg. The average blood pressure of the positive control group increased 6 h after administration. This tendency of reduced blood pressure in SHR 4 h after oral administration was similar to that of the commercial antihypertensive drug, Captopril. This suggests that the water extracts containing ACE inhibitor from *H. marmoreus* fruiting body has a clear antihypertensive effect in SHRs; at a dosage of 800 mg/kg.

In conclusion, the antihypertensive ACE inhibitor in water extracts from *Hypsizygus marmoreus* (brown cultivar) fruiting body was purified with ultrafiltration, C_18_ solid phase extraction chromatography, and reverse-phase HPLC. A purified ACE inhibitor with an inhibitory activity (IC_50_) of 0.19 mg/mL was obtained. The purified ACE inhibitor was found to be an oligo-peptide with the sequence LSMGSASLSP. Its molecular weight was estimated to be 567.3 Da, and the water-extract containing ACE inhibitor showed clear antihypertensive effect on a spontaneously hypertensive rat.

## Figures and Tables

**Table 1:** Comparison of sequence and ACE inhibitory activity between ACE inhibitory peptides from some mushrooms and *Hypsizygus  marmoreus *in this study.

Mushrooms	Peptide	Molecular weight (Da)	IC_50_(mg/mL)^a^	References
*Pleurotus cornucopiae *	RLPSEFDLSAFLRA	1622.85	0.460	Jang et al. 2011 [11]
	RLSGQTIEVTSEYLFRH	2037.26	1.140	
*Pholiota adiposa *	GOGGP	414.00	0.044	Koo et al. 2006 [10]
*Tricholoma giganteum *	GOP	301.10	0.040	Lee et al. 2004 [9]
*Hypsizygus marmoreus *	LSMGSASLSP	567.30	0.190	This study

Captopril			17.9 nM	commercial drug

^a^The concentration of an ACE inhibitor required to inhibit 50% of ACE activity.
